# Neurodevelopmental Disorder Traits in *Taijin-Kyofu-sho* and Social Anxiety Disorder: A Cross-Sectional Study among University Students

**DOI:** 10.1155/2021/1661617

**Published:** 2021-09-17

**Authors:** Kosuke Kajitani, Rikako Tsuchimoto, Yusaku Omodaka, Tomoko Matsushita, Hideaki Fukumori, Takeshi Sato, Jun Nagano

**Affiliations:** Center for Health Sciences and Counseling, Kyushu University, 744, Motooka, Nishi-ku, Fukuoka 819-0395, Japan

## Abstract

*Taijin-Kyofu-sho* is an East Asian culture-bound anxiety disorder with similarities to social anxiety disorder. However, few studies have examined these two disorders from the perspective of neurodevelopmental disorders. This study is aimed at examining the association of *Taijin-Kyofu-sho* and social anxiety disorder with the attention-deficit/hyperactivity disorder (ADHD) trait and autism spectrum disorder (ASD) trait. The Liebowitz Social Anxiety, *Taijin-Kyofu-sho*, and Adult Attention-Deficit/Hyperactivity Disorder Self-Report scales and the 16-item Autism-Spectrum Quotient were administered to 818 university students. Participants were divided into four groups: control (neither *Taijin-Kyofu-sho* nor social anxiety disorder), pure *Taijin-Kyofu-sho* (*Taijin-Kyofu-sho* alone), pure social anxiety disorder (social anxiety disorder alone), and mixed *Taijin-Kyofu-sho*-social anxiety disorder (both *Taijin-Kyofu-sho* and social anxiety disorder). We used logistic regression analysis to examine whether the ADHD trait and ASD trait were associated with *Taijin-Kyofu-sho* and social anxiety disorder. ASD trait was significantly associated with pure *Taijin-Kyofu-sho* (*p* = 0.006, odds ratio: 3.99). Female sex and ADHD trait were significantly associated with pure social anxiety disorder (sex: *p* = 0.013, odds ratio: 2.61; ADHD trait: *p* = 0.012, odds ratio: 2.46). Female sex, ADHD trait, and ASD trait were significantly associated with mixed *Taijin-Kyofu-sho*-social anxiety disorder (sex: *p* = 0.043, odds ratio: 2.16; ADHD trait: *p* = 0.003, odds ratio: 2.75; ASD trait: *p* < 0.001, odds ratio: 16.93). Neurodevelopmental disorder traits differed between individuals with *Taijin-Kyofu-sho* and those with social anxiety disorder. Overall, our study reveals that Japanese individuals with the ADHD or ASD traits are at a heightened risk of developing *Taijin-Kyofu-sho* or social anxiety disorder.

## 1. Introduction

Neurodevelopmental disorders (NDDs) result from impairments in brain growth and development that lead to emotional and behavioral problems that begin in early childhood and persist into adulthood. According to the Diagnostic and Statistical Manual of Mental Disorders, Fifth Edition (DSM-5), NDDs are characterized by developmental deficits of varying severity in personal, academic, or occupational functioning [1]. Among NDDs, autism spectrum disorder (ASD) and attention-deficit/hyperactivity disorder (ADHD) often cause significant problems for college students. Students with ADHD or ASD are at risk of poor academic achievement and social isolation and are less likely to pursue a college education [2–4]. Furthermore, in young adults, ADHD or ASD is often comorbid with other psychiatric disorders, including anxiety disorder, mood disorder, and obsessive-compulsive disorder [5–7], which worsens their social adaptation [8]. In fact, having a mental health comorbidity is a negative predictor of quality of life for adults with ASD [9], and complications of psychiatric disorders are associated with poor academic performance in children with ADHD [10]. Thus, it is important to consider how comorbid disorders affect the quality of life of students with NDDs.

Social anxiety disorder (SAD) is an anxiety disorder characterized by marked fear of social situations in which the individual is exposed to possible scrutiny by others [11]. Examples of social situations include social interaction (e.g., having a conversation and meeting unfamiliar people), being observed (e.g., eating or drinking), and performing in front of others (e.g., giving a speech). Accumulating evidence suggests that patients with NDDs often have SAD symptoms. For example, Freeth et al. examined the association between ASD and SAD using the Autism-Spectrum Quotient (AQ) and Liebowitz Social Anxiety Scale (LSAS) and indicated that British students with multiple autistic traits were more likely to report increased social anxiety than those with few autistic traits [12]. A systematic review by Spain et al. on the relationship between ASD symptoms and SAD in individuals with ASD found that SAD symptoms were associated with poorer social skills and functioning [13]. Additionally, studies have reported high rates of ADHD in adult patients with SAD [14, 15]. In particular, the presence of comorbid ADHD has been associated with increased severity and functional impairment on the Global Assessment of Functioning Scale [16]. Thus, the relationship between SAD and ADHD/ASD has been studied in terms of epidemiology and social function.

SAD was previously known as “social phobia,” a term coined by Janet in the early 1900s [17]. However, the concept of SAD did not exist in the U.S. until Marks and Gelder differentiated social phobia from phobic disorders in the 1960s [18]. Meanwhile, in Japan, SAD has been reported since the 1920s. Morita initially termed the features of SAD as *Taijin-Kyofu-sho* (TK), which literally means disorder (*sho*) of fear (*kyofu*) of interpersonal relations (*taijin*) [19, 20]. TK is defined as persistent and excessive fear of offending others in social situations with one's physical characteristics, such as blushing, gaze, or body odor [21]. It has been considered a subtype of neurosis or neurasthenic state, which is called *Shinkeishitsu* in Japanese [20] and is regarded as a culture-bound anxiety disorder in Japan and other East Asian countries [22]. In the DSM-5, TK is listed under SAD and briefly described in the section on culture-related diagnostic issues [11].

The psychopathologies of TK and SAD have been compared. There are two types of TK: “tension” and “offensive” [23]. In the tension type, patients fear being looked down upon in social situations because of a physical manifestation of anxiety or embarrassment, such as blushing. They also feel ashamed for experiencing these anxieties and fears and therefore avoid anxiety-provoking social situations. These features resemble the “fear of being noticed” seen in patients with SAD. Furthermore, Nakamura et al. compared the clinical diagnosis of TK and the operational diagnosis of SAD according to the DSM-IIIR. They found that 65.8% of patients with TK were also diagnosed with SAD [24], suggesting that TK and SAD share clinical and psychopathological features.

The offensive type of TK is characterized by fear of offending or disgusting others due to eye contact or body odor. Patients with offensive type TK believe that they are offending others with their physical appearance or that others are avoiding them [20]. Previously, the major difference between TK and SAD was considered to be that patients with TK fear offending others, whereas patients with SAD fear embarrassing themselves [25]. Therefore, TK has been described as an other-oriented (allocentric) disorder and SAD as a self-oriented (egocentric) disorder [26].

With the publication of the DSM-5, the differences between the features of TK and SAD have become ambiguous. The following sentence was added to the DSM-5 as “criterion B” of SAD: “The individual fears that he or she will act in a way or show anxiety symptoms that will be negatively evaluated (i.e., will be humiliating or embarrassing; will lead to rejection or offend others)” [11]. The definition of SAD was expanded because TK has been observed not only in East Asians but also in individuals from Western countries [27, 28]. However, some subcategories of TK are difficult to classify into the DSM-5 criteria of SAD (e.g., *Shubo-kyofu*, the phobia of a deformed body, and *Jikoshu-kyofu*, the phobia of one's own foul body odor) [25, 29]. Thus, although TK has been regarded as a type of SAD, it is still controversial whether these two disorders are identical because of several differences in their clinical features.

Since TK and SAD are unlikely to be identical in some respects, we considered it useful to focus on the differences in the causes of these two anxiety disorders. To examine the differences in psychopathology between TK and SAD, we previously compared SAD and TK in terms of ADHD traits [30]. However, our previous study did not compare TK and SAD with respect to other NDDs. Here, we hypothesized that the ADHD and ASD traits may contribute to the onset of TK and SAD. This study is aimed at examining the association of TK and SAD with the ADHD and ASD traits in university students.

## 2. Materials and Methods

### 2.1. Study Design and Subjects

This cross-sectional study was conducted at Kyushu University from April 2015 to April 2018. All participants were Japanese students at the Department of Interdisciplinary Graduate School of Engineering Sciences and Faculty of Arts and Science at Kyushu University who volunteered to participate in the study. We obtained informed consent from the students as follows: students were provided with our research manual and received an oral explanation of the study (purpose, methods, risk, and the right to withdraw from the study). We then asked students enrolled in psychology, health, and safety courses to volunteer. The questionnaires were completed anonymously, and the answers did not contain any identifying information. Submission of the questionnaire was regarded as consent for participation in the study. Furthermore, the participants were provided opportunities to opt-out from the study. Students who had previously been administered the scales used in the study were excluded to avoid duplication. This study was approved by the Ethics Committee of the Faculty of Arts and Science, Kyushu University, Japan (approval number: 201406R).

### 2.2. Measures

#### 2.2.1. The Taijin-Kyofu-sho Scale

The *Taijin-Kyofu-sho* scale (TKs), developed by Kleinknecht et al. in 1997, consists of 31 items related to TK symptoms that best differentiate patients with TK from controls [19]. Participants were instructed to rate all 31 items on a seven-point scale (1 = totally false to 7 = exactly true), with total TKs scores ranging from 31 to 217. Based on the study by Kleinknecht et al., participants scoring 122 or higher were considered to have TK [19, 30].

#### 2.2.2. The Liebowitz Social Anxiety Scale (LSAS)

We used the Japanese version of the LSAS in this study [31]. The LSAS consists of 24 items that assess the extent of social interactions and performance in situations that may trigger SAD symptoms [32]. Participants rated all items on a four-point scale (0 = none to 3 = severe) based on the fear felt during specific situations (fear or anxiety section), following which they were rated again based on the avoidance of a situation (avoidance section). Total fear/anxiety and total avoidance scores ranged from 0 to 72 each; thus, the total LSAS score ranged from 0 to 144. Based on previous reports, participants scoring 60 or higher were considered to have SAD [33–36].

#### 2.2.3. The Adult Attention-Deficit/Hyperactivity Disorder Self-Report Scales

The ASRS version 1.1 is a self-report questionnaire designed to screen for adult ADHD [37]. It consists of 18 items rated on a five-point scale (0 = never to 4 = very often). Six of the questions (part A: items 1 to 6) are reported to be predictive of symptoms consistent with ADHD and are often used to define adult ADHD. Each item has a minimum score allowing a “positive” classification (2 (sometimes) or 3 (often)), and the total number of “positive” items is counted. The minimum number of positive ASRS-6 part A items for the indication of adult ADHD is 4. Accordingly, we adopted a cut-off value (COV) of 4 for the definition of the ADHD trait in this study (ASRS − 6 ≥ 4: ADHD +; <4: ADHD −).

#### 2.2.4. The 16-Item Autism-Spectrum Quotient (AQ16)

The AQ, developed by Baron-Cohen et al. [38], is a 50-item self-report questionnaire that measures autistic traits. The AQ uses a four-point Likert-type scale (strongly agree, slightly agree, slightly disagree, and strongly disagree). Slightly or strongly agree responses are each scored 1 point, resulting in a total score ranging from 0 to 50. Although it is widely used to measure autism spectrum tendencies in clinical research, the 50-item version of the AQ is too lengthy to be included in comprehensive screening for psychiatric disorders. Therefore, shorter versions of the AQ have been developed as screening tools for ASD. To examine the extent of autistic traits in participants, we used the Japanese version of the 16-item Autism-Spectrum Quotient (AQ16), which was designed to detect Asperger's syndrome [39]. According to previous research, a COV of 12 on the AQ16 (score range: 0 to 16) has high sensitivity (0.80) and specificity (0.97) [39], and we therefore adopted a COV of 12 points for the definition of the ASD trait in this study (AQ16 ≥ 12: ASD +; <12: ASD −).

### 2.3. Data Analysis

First, we classified the participants into TK+ and TK− groups based on their TKs scores (TK+ met the abovementioned criteria for TK; TK− did not meet the abovementioned criteria for TK). Similarly, they were classified into SAD+/SAD−, ADHD+/ADHD−, and ASD+/ASD− groups according to the relevant definitions. Second, we compared the prevalence of TK and SAD across the four groups based on ADHD trait/ASD trait, that is, ADHD− ASD−, ADHD+ ASD−, ADHD− ASD+, and ADHD+ ASD+. Third, we used multiple logistic regression analysis to examine the potential contributions of ADHD trait and ASD trait to the occurrence of TK or SAD, with TK (− = 0, + = 1) or SAD (− = 0, + = 1) as the dependent variable, ADHD trait (− = 0, + = 1) and ASD trait (− = 0, + = 1) as independent variables, and age (years) and sex (male = 0, female = 1) as common covariates. Finally, because some participants had comorbid TK and SAD, we attempted to characterize them based on the differences in symptoms associated with TK and SAD. For this purpose, we further classified them into “pure TK,” “pure SAD,” and “TK-SAD mixed,” where an individual with pure TK only met the criteria for TK but not for SAD (the opposite was true for pure SAD), and an individual with TK-SAD mixed met the criteria for both TK and SAD. We then repeated logistic regression analyses with pure TK, pure SAD, and TK-SAD mixed as dependent variables. The chi-square test or Fisher's exact test was used for comparing categorical variables between groups; the Kruskal-Wallis test was used for the other variables. Data were analyzed using SPSS version 27.0 (IBM Corporation, Armonk, NY, USA). Statistical tests were two-sided, and *p* values < 0.05 were considered significant.

## 3. Results

**Figure 1:**
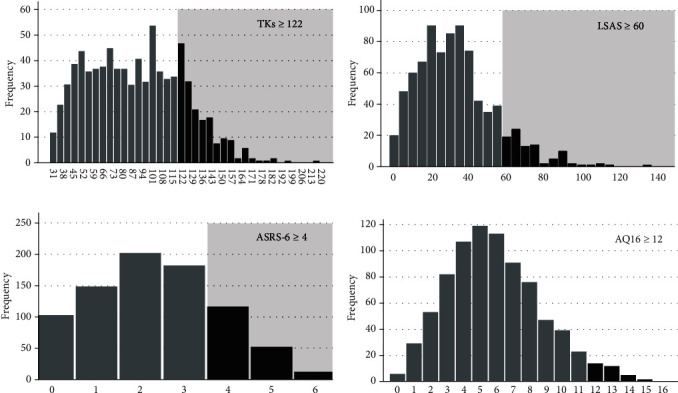
Histograms of the psychological test scores. The horizontal axes indicate the scores for each test. The vertical axes indicate the number of participants (frequency) with each score. The gray area indicates participants whose scores exceeded the cut-off value (COV). (a) Distribution of the *Taijin-Kyofu-sho* scale (TKs) scores. COV: 122. (b) Distribution of the Liebowitz Social Anxiety Scale (LSAS) scores. COV: 60. (c) Distribution of the adult Attention-Deficit/Hyperactivity Disorder Self-Report Scale-6 (ASRS-6) scores. COV: 4. (d) Distribution of the 16-item Autism-Spectrum Quotient (AQ16) scores. COV: 12.

In total, 818 students (males: 628, females: 190) completed all the psychological tests. The mean age (standard deviation (SD): range) of the participants was 21.4 years (2.02: 18–40 years).  Figure 1 shows the frequency distributions of the TKs (a), LSAS (b), ASRS-6 (c), and AQ16 (d) scores; the corresponding COVs are shown in each graph. Of the 818 participants, 150 (18.3%), 86 (10.5%), and 47 (5.7%) were classified as pure TK, pure SAD, and TK-SAD mixed, respectively. With respect to NDDs, 182 (22.2%), 33 (4.0%), and 20 (2.4%) had ADHD trait, ASD trait, and comorbid ADHD trait and ASD trait, respectively. The mean (SD) TKs, LSAS, ASRS-6, and AQ16 scores were 89.0 (33.7), 32.9 (20.7), 2.33 (1.48), and 5.87 (2.86), respectively.

The characteristics of the participants are shown in Table 1. The participants with TK or SAD were slightly younger, were more likely to be female, and had a higher proportion of comorbid ADHD trait or ASD trait than controls.

**Figure 2:**
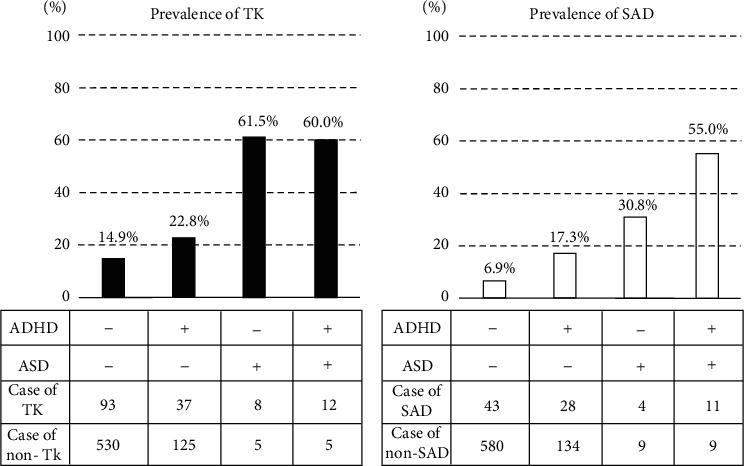
Prevalence of *Taijin-Kyofu-sho* and social anxiety disorder in participants with neurodevelopmental disorder traits. Participants were divided into four groups (ADHD – ASD –, ADHD + ASD –, ADHD –ASD +, and ADHD + ASD +) to examine the groupwise prevalence of TK and SAD. (a) Black bars indicate the prevalence of TK. (b) White bars indicate the prevalence of SAD. ADHD: attention-deficit/hyperactivity disorder; ASD: autism spectrum disorder; TKs: *Taijin-Kyofu-sho*; SAD: social anxiety disorder.

Figure 2 shows the prevalence of TK (a) and SAD (b) across the groups based on ADHD trait/ASD trait, that is, ADHD− ASD−, ADHD+ ASD−, ADHD− ASD+, and ADHD+ ASD+. The proportion (number) of participants with TK was 14.9% (93/623), 22.8% (37/162), 61.5% (8/13), and 60.0% (12/20) in the ADHD– ASD–, ADHD+ ASD−, ADHD– ASD+, and ADHD+ ASD+ groups, respectively. The proportion (number) of participants with SAD was 6.9% (43/623), 17.3% (28/162), 30.8% (4/13), and 55.0% (11/20) in the ADHD– ASD–, ADHD+ ASD−, ADHD– ASD+, and ADHD+ ASD+ groups, respectively. Thus, the prevalence of TK/SAD differed based on the presence/absence of ADHD trait/ASD trait, and the pattern of association with ADHD trait/SD trait seemed to be substantially different between TK and SAD. Indeed, there was a statistically significant difference in the prevalence of TK and SAD among the groups ((a) *p* < 0.001, (b) *p* < 0.001, Fisher's exact test).

As shown in Figure 2, the presence of ADHD trait/ASD trait resulted in differences in the proportions of TK and SAD. To confirm whether ADHD trait and ASD trait were associated with TK and SAD, logistic regression analyses were conducted with TK or SAD (0 = control, 1 = TK or SAD) as a binary outcome and age, sex, ADHD trait, and ASD trait as variables. As shown in Table 2, among the four variables, ADHD trait and ASD trait were significantly associated with TK (ADHD trait: *p* = 0.032, odds ratio (OR) 1.57; ASD trait: *p* < 0.001, OR 6.63). Female sex, ADHD trait, and ASD trait were significantly associated with SAD (female sex: *p* = 0.003, OR 2.29; ADHD trait: *p* < 0.001, OR 2.72; ASD trait: *p* < 0.001, OR 6.46).

We found that ADHD trait and ASD trait were both significantly associated with TK and SAD (Table 2). However, these analyses included 47 cases of comorbid TK and SAD. The characteristics of the participants grouped according to the presence or absence of TK and SAD are shown in Table 3. ASD was significantly associated with pure TK (ASD: *p* = 0.006; OR 3.99). Female sex and ADHD trait were significantly associated with pure SAD (sex (female): *p* = 0.013, OR 2.61; ADHD trait: *p* = 0.012, OR 2.46). Female sex, ADHD trait, and ASD trait were significantly associated with TK-SAD mixed (sex (female): *p* =0.043, OR 2.16; ADHD trait: *p* = 0.003, OR 2.75; ASD trait: *p* < 0.001, OR 16.93) (Table 4).

## 4. Discussion

In this study, we found that a higher proportion of participants with TK and SAD had ADHD trait and ASD trait than those in the control group. Using logistic regression analysis, we identified ADHD trait and ASD trait as factors associated with TK. In addition to ADHD trait and ASD trait, female sex was significantly associated with SAD. Furthermore, to highlight the differences between the clinical features of TK and SAD, we divided the participants into four groups (control, pure TK, pure SAD, and TK-SAD mixed) and performed logistic regression analyses. ASD trait was associated with pure TK, and female sex and ADHD trait were associated with pure SAD. We believe this is the first study comparing TK and SAD according to NDDs (ADHD and ASD). Our results may provide new insights into the pathogenesis of TK and SAD, which can contribute to the early management of these anxiety disorders.

Although few studies have examined the prevalence of TK among patients with developmental disorders, many have shown a relationship between SAD and NDDs in adults. Studies from the U.S. and Norway have reported that 29.3% [40] and 14.2% [41] of adults with ADHD had comorbid SAD, respectively. Here, 21.4% (39/182) of participants with ADHD trait had SAD, which was in line with the results of previous reports.

Adults with ASD have been reported to have a significantly higher prevalence of SAD than those without ASD (21.7% vs. 5.0%) [42]. Furthermore, a systematic review estimated the prevalence of SAD in adults with ASD as 29% [43]. However, we observed a higher prevalence of SAD in participants with ASD trait (45.5% (15/33)) than that in previous reports. This may be explained by differences in the participants' backgrounds or the evaluation method used. Our research focused on university students, whereas other studies included participants with various backgrounds (e.g., education, occupation, economic status, and race). Moreover, participants of previous studies were clinically diagnosed with ASD based on the DSM or International Classification of Diseases criteria, in contrast to the self-administered test (AQ16) used in our study.

Logistic regression analysis identified ADHD trait and ASD trait as common factors associated with TK and SAD. However, the analysis included 47 cases of comorbid TK and SAD. To exclude the effect of TK and SAD on each other, we divided the participants into pure TK and pure SAD groups and performed logistic regression analysis. As a result, ASD trait was identified as a factor associated with pure TK, and ADHD trait and female sex were identified as factors associated with pure SAD.

One possible reason for the association between ASD trait and TK is collectivism. Contrary to individualism in Western countries, East Asian societies, including Japan, are characterized by collectivism. In collectivism, people need to sensitively perceive the feelings or thoughts of others because “not bothering others” and “cooperativeness” are regarded as virtues; that is, the fear of being disliked and not accepted plays a prominent role in collectivistic societies like Japan [44]. However, patients with ASD have deficits in empathy and “theory of mind,” and they often have difficulties in understanding the thoughts of others and communicating with them properly [45, 46]. Therefore, patients with ASD are unable to form and maintain positive peer relationships, and some of them are even bullied in childhood. Indeed, there is a growing body of literature indicating that patients with ASD are at a significantly higher risk of both victimization and perpetration [47]. It is possible that these interpersonal failures and painful experiences may make patients with ASD oversensitive to communication with others; that is, some patients with ASD may always worry that their words and actions may hurt others' feelings or make them uncomfortable. This anxiety about “making peers uncomfortable” is a core feature of TK.

Another reason for the association between ASD trait and TK may be social withdrawal. Social withdrawal, a maladaptive behavior associated with ASD, is more prevalent among children with ASD than typically developing children and often worsens with age [48]. Meanwhile, patients with severe TK are often socially withdrawn. This state is called *Hikikomori*, which literally means “pulling inward” in Japanese. The definition of *Hikikomori* is abnormal avoidance of social contact, typically by adolescents [49]. Indeed, a case series of inpatients with TK revealed that 30% of them could be regarded as *Hikikomori* [50]. Moreover, using the AQ, Katsuki et al. have shown that patients with *Hikikomori* were more likely to have autistic tendencies than non-*Hikikomori* individuals [51]. Thus, social withdrawal in TK and *Hikikomori*, which was first described in Japan, seems to be closely related to ASD.

Accumulating evidence suggests that SAD is closely associated with ADHD. Koyuncu et al. reported that the rate of childhood ADHD in SAD was high (72.3%) [16]. In a cross-sectional observational study, Sanz et al. found that adolescents with inattentive ADHD had a higher degree of SAD [52]. Furthermore, Nelson and Liebel demonstrated that college students with ADHD have significantly more anxiety symptoms than those without ADHD [53]. In their report, participants with inattentive ADHD had more anxiety symptoms than in those with combined ADHD (inattentive and hyperactive-impulsive ADHD). Thus, considering the close relationship between SAD and ADHD, it is not surprising that ADHD trait is associated with pure SAD.

Previous studies have consistently shown that women are more likely to have SAD, to have more severe clinical symptoms of SAD, and to have greater subjective distress compared to men [54, 55]. Here, consistent with their results, we found that female sex was associated with pure SAD. However, sex differences in TK remain controversial. TK reportedly has a male predominance of approximately 3 : 2 [17]. However, Maeda and Nathan reported that the proportion of female patients with TK was increasing [20]. Furthermore, Essau et al. compared British and Japanese young adults using the TKs and found that British females had significantly higher scores than males; however, no significant sex differences were found in a previous analysis of the Japanese population [56]. We found no sex differences in pure TK on logistic regression analysis. Epidemiological studies with larger samples are needed to examine the sex differences in TK.

### 4.1. Limitations of This Study

This is the first study to examine the association between two types of anxiety disorders (TK and SAD) and developmental disorders (ADHD and ASD) using logistic regression analysis. However, it has some limitations. First, all the participants were recruited from a single university, and 70% of the students were male. Moreover, Kyushu University is ranked as one of the top 10 universities in Japan, due to which the intelligence level of its students is expected to be relatively high. Therefore, our results cannot be generalized to all Japanese young adults. Second, we relied on self-reported assessments to diagnose TK, SAD, ADHD, and ASD. In general, a clinical diagnosis should be made according to the operational diagnostic criteria based on a psychiatric interview. Therefore, a clinical evaluation using structured interviews is required for the accurate diagnosis of these disorders. We also emphasize that our study investigated the traits of each disorder and did not intend to diagnose any of these disorders. Moreover, the TK was developed to evaluate the extent of TK symptoms, and further research is required to ascertain whether it is suitable for diagnosing TK.

## 5. Conclusions

In this study, we examined the association between anxiety disorders (TK and SAD) and the ADHD and ASD traits. Multivariate logistic regression analyses revealed that ASD was significantly and independently associated with TK, and ADHD trait and female sex were significantly and independently associated with SAD. Differences in ADHD and ASD traits may influence the future onset of TK or SAD in Japanese adults.

## Figures and Tables

**Table 1:** Characteristics of participants.

	Control (TKs < 122, LSAS < 60)	Participants with TK (TKs ≥ 122)	Participants with SAD (LSAS ≥ 60)	*p* value
Number	629	150†	86†	—
Mean age (standard deviation)	21.4 (2.01)	21.1 (2.01)	20.7 (2.16)	0.008^+^
Sex, F/M (% F)	129/500 (20.5%)	44/106 (29.3%)	36/50 (41.9%)	*p* < 0.001^#^
ADHD trait (%)	118 (18.8%)	49 (32.7%)	39 (45.3%)	*p* < 0.001^#^
ASD trait (%)	11 (1.7%)	20 (13.3%)	15 (17.4%)	*p* < 0.001^#^

TK: *Taijin-Kyofu-sho*; SAD: social anxiety disorder; TKs: *Taijin-Kyofu-sho* scale; LSAS: Liebowitz Social Anxiety Scale; M: male; F: female; ADHD: attention-deficit/hyperactivity disorder; ASD: autism spectrum disorder. †There were 47 cases of comorbid TK and SAD. ^+^*p* < 0.01, Kruskal-Wallis test; ^#^*p* < 0.001, chi-square test.

**Table 2:** Factors associated with TK and SAD on logistic regression analysis.

Factor	TK		SAD	
	OR (95% CI)	*p* value	OR (95% CI)	*p* value
Age	0.97 (0.87–1.07)	0.48	0.90 (0.79–1.03)	0.12
Female sex	1.37 (0.87–2.15)	0.17	*2.29 (1.33–3.96)*	0.003
ADHD trait	*1.58 (1.04–2.39)*	0.032	*2.72 (1.66–4.46)*	< 0.001
ASD trait	*6.63 (3.15–13.98)*	< 0.001	*6.46 (2.93–14.25)*	< 0.001

Italic text indicates statistical significance. TK: *Taijin-Kyofu-sho*; SAD: social anxiety disorder; OR: odds ratio; CI: confidence interval; ADHD: attention-deficit/hyperactivity disorder; ASD: autism spectrum disorder.

**Table 3:** Characteristics of participants classified according to TKs and LSAS cut-off values.

	Control LSAS (< 60)/TKs (<122)	Pure TK LSAS (< 60)/TKs (≥122)	Pure SAD LSAS (≥60)/TKs (<122)	TK-SAD mixed LSAS (≥60)/TKs (≥122)	*p* value
Number	629	103	39	47	
Mean age (SD)	21.4 (2.01)	21.3 (1.81)	20.7 (1.92)	20.7 **(**2.37)	0.03^a^^∗^
Sex, F/M (% F)	129/500 (20.5%)	25/78 (24.3%)	17/22 (43.6%)	19/28 (40.4%)	< 0.001^b^^∗∗^
Mean ASRS-6 score (SD)	2.19 (1.45)	2.52 (1.36)	2.74 (1.43)	3.45 (1.56)	< 0.001^a^^∗∗^
Mean AQ16 score (SD)	5.37 (2.60)	7.09 (2.83)	6.85 (2.59)	9.40 (2.92)	< 0.001^a^^∗∗^

TK: *Taijin-Kyofu-sho*; SAD: social anxiety disorder; LSAS: Liebowitz Social Anxiety Scale; TKs: *Taijin-Kyofu-sho* scale; M: male; F: female; SD: standard deviation; ASRS-6: Attention-Deficit/Hyperactivity Disorder Self-Report Scale-6; AQ16: 16-item Autism-Spectrum Quotient (AQ16). ^a^Kruskal-Wallis test; ^b^chi-square test; ^∗^*p* < 0.05, ^∗∗^*p* < 0.001.

**Table 4:** Factors associated with pure TK, pure SAD, and TK-SAD mixed on logistic regression analysis.

	Pure TK		Pure SAD		TK-SAD mixed	
	OR (95% CI)	*p* value	OR (95% CI)	*p* value	OR (95% CI)	*p* value
Age	0.98 (0.87–1.10)	0.73	0.91 (0.75–1.09)	0.309	0.86 (0.72–1.03)	0.11
Female sex	1.24 (0.73–2.11)	0.43	*2.61 (1.23–5.55)*	*0.013*	*2.16 (1.03–4.56)*	*0.043*
ADHD trait	1.26 (0.76–2.09)	0.38	*2.46 (1.22–4.97)*	*0.012*	*2.75 (1.40–5.42)*	*0.003*
ASD trait	*3.99 (1.47–10.78)*	*0.006*	3.82 (0.54–14.68)	0.22	*16.93 (6.37–45.01)*	*< 0.001*

Italic text indicates statistical significance. TK: *Taijin-Kyofu-sho*; SAD: social anxiety disorder; OR: odds ratio; CI: confidence interval; ADHD: attention-deficit/hyperactivity disorder; ASD: autism spectrum disorder.

## Data Availability

The data used in this study are available from the corresponding author upon request.
